# The relationship between childhood adversity and problem behavior of new street corner youth on campus: A moderate mediation model

**DOI:** 10.3389/fpsyg.2022.1036773

**Published:** 2022-11-17

**Authors:** LinLin Fan, WeiJie Meng

**Affiliations:** ^1^School of Educational Science, Ludong University, Yantai, China; ^2^Institute for Education and Treatment of Problematic Youth, Ludong University, Yantai, China

**Keywords:** new street corner youth on campus, problem behavior, childhood adversity, life history strategy, dark triad, adolescent

## Abstract

To explore the impact mechanism of childhood adversity on problem behaviors of new street corner youth on campus, we surveyed 637 new street corner youth on campus and completed the Strengths and Difficulties Questionnaire, the Childhood Environment Scale, the Life History Strategies Scale, and the Dark Triad Scale. After controlling for gender and age of new street corner youth on campus, results revealed that childhood adversity was significantly and positively associated with problem behaviors. Mediation analysis showed that life history strategy mediated the association between childhood adversity and problem behaviors. Moreover, moderated mediation analysis further indicated that dark triad moderated the association between childhood adversity and life history strategy, as well as the association between life history strategy and problem behaviors. These findings suggest that interventions of life history strategy and dark triad may be effective means to affect problem behaviors of new street corner youth on campus.

## Introduction

Previous studies have found that street corner youth frequently engage in violent behaviors (Baron, 2003), crime (Baron and Forde, 2007), drug use (Wansi et al., 1996), and alcohol abuse (Hadlanda et al., 2009), and that they are deviant and delinquent in nature (Karabanow and Clement, 2004). Fu (2003) believes that students have interactions with street corner youth and refers to this group on campus that interacts with street corner youth as the new street corner youth on campus. The study found that students in long-term interactions with street corner youth had many problem behaviors, with 46% of them reporting refusal to attend school and 47% absenteeism from school, and were associated with delinquent behavior (Olley, 2006). Therefore, the problem behaviors of new street corner youth on campus need to be paid attention to by researchers.

Problem behaviors in adolescents have received extensive attention from researchers because of their prevalence, severity, and persistence (Burt and Roisman, 2010). Problem behavior (PB) refers to abnormal behaviors in individuals that hinder their social adjustment, both in terms of their own emotional abnormalities and behaviors that negatively affect others and society (Zhang, 2019). Problem behaviors consist of both internalized problem behaviors such as anxiety and depression, and externalized problem behaviors such as inattention and aggression (Chao et al., 2018). The measurement of problem behaviors in China has mostly used the Strengths and Difficulties Questionnaire developed by Kou et al. (2007), which categorizes problem behaviors into four dimensions, namely, emotional symptoms, conduct problems, hyperactivity, and peer interaction problems, summarizing internalizing and externalizing problem behaviors. A related study indicated that more than 60% of individuals develop problem behaviors during adolescence (Van et al., 2020). About 30 million children and adolescents in China have different levels of problem behaviors, and this figure is still increasing year by year (Chi et al., 2021). Problem behaviors not only have a greater negative impact on adolescents’ physical and mental development, academics, and interpersonal interactions (Liu et al., 2018), but also jeopardize their later socialization development, and in serious cases, even lead adolescents to delinquency (Yap and Jorm, 2015). Therefore, the prevention and intervention of problem behaviors in adolescents has been a key topic of interest for researchers. The present study explores the influencing factors and mechanisms of problem behaviors in order to propose effective strategies for the prevention of problem behaviors of new street corner youth on campus.

### Childhood adversity and adolescents problem behaviors

Life-course theory divides the life course into childhood, adulthood, and old age, emphasizes the influence of early factors, believes that the results of human behavior should be traced back to the “upstream” factors of the life course, and the early life course is the most critical impact on the individual, which lasts for a lifetime and is difficult to remedy or eliminate (Bernardia et al., 2019). Childhood adversity (CA) refers to stressful or traumatic experiences that occur during childhood, including physical or emotional abuse during childhood and chronic environmental stress (Dube et al., 2001). A previous study found that childhood adversity is associated with adolescent violence, and individuals who experienced abuse and violence in childhood are more likely to be violent in adolescence and early adulthood (Salo et al., 2021). A specific study using a life-course framework to examine the impact of adverse childhood experiences with internalizing and externalizing problems found that early adverse experiences were significantly associated with problem behaviors (Schroeder et al., 2020). Similar results were found in related studies of Chinese adolescents, where adolescents with more difficult and unpredictable childhood environments had more internalizing and externalizing problem behaviors (Chang and Lu, 2018; Chang et al., 2021). Therefore, we propose:

*Hypothesis1*: there is a significant positive association between childhood adversity and problem behaviors of new street corner youth on campus.

### The mediating role of life history strategy

Life history strategy (LHS) refers to a kind of allocation and trade-off strategy of human beings for various resources. This theory holds that in order to survive and reproduce, individuals will allocate and trade off their own resources when facing limited resource conditions (Gladden et al., 2010). Researchers describe life history strategies as a continuum from “fast” to “slow,” with faster life history strategies associated with earlier age of fertility and sexual development and a preference for immediate satisfaction. Conversely, slower life history strategies are associated with later fertility and sexual development, and a preference for delayed satisfaction (Figueredo et al., 2005; Bielby et al., 2007; Griskevicius et al., 2013). Individuals’ choice of life history strategies is related to early life experiences (Belsky et al., 1991; Kuzawa et al., 2010). Individuals are more inclined to adopt fast life history strategy when their early childhood environment is more difficult and unpredictable (White et al., 2013). Due to the instability of the living environment, individuals are exposed to many risk factors, and using a slow life history strategy will expose individuals to greater risk. Therefore, fast life history strategies are meaningful in harsh and unpredictable ecological environments. In conclusion, the harshness and unpredictability of the environment will motivate individuals to choose a fast life history strategy. Conversely, when the life environment is stable and controllable, individuals prefer a slow life history strategy (Ellis et al., 2009).

Different life history strategy leads to different behavioral outcomes. Individuals who use fast life history strategy pay more attention to the moment, emphasize short-term effects, and are more inclined to take impulsive risk-taking, instant gratification, and even social irregularities; Individuals who use the slow life history strategy pay more attention to long-term development, tend to delay gratification, and obey the law (Figueredo et al., 2004, 2005). Minkov and Beaver (2016) analyzed worldwide indices of criminal violence (homicide, robbery, and assault) and showed that differences in criminal violence in most countries are largely due to differences in life history strategy, and they argue that life history theory is the most compelling explanation for violent crime as a complex phenomenon. Recent research examining the relationship between childhood circumstances, life history strategy, and problem behaviors among Chinese adolescents found that adolescents with more difficult and unpredictable childhood circumstances tended to choose fast life history strategy and tended to have more aggressive behaviors, impulsivity, poor academic performance, internalized and externalized problem behaviors (Chang and Lu, 2018; Chang et al., 2019; Lu and Chang, 2019; Chang et al., 2021). Therefore, we propose:

*Hypothesis 2*: life history strategy mediates the relationship between childhood adversity and problem behaviors of new street corner youth on campus.

### The moderating effect of dark triad

Dark triad (DT) refers to a group of personality traits consisting of three dark personalities: Machiavellianism, psychopathy, and narcissism, with common manifestations such as aggressive behavior, lack of empathy, and disregard for conventional morality (Paulhus and Williams, 2002). Narcissism is characterized by an exaggerated self-concept, an excessive sense of self, an overwhelming sense of self-worth, and a general lack of concern for others (Vonk et al., 2013). Machiavellianism is characterized by manipulativeness, control, and deception for personal gain (Esperger and Bereczkei, 2012). The main characteristics of psychopathy include apathy, stimulus-seeking behavior, and impulsivity (Brankley and Rule, 2014).

Dark triad may moderate the process by which childhood adversity affects life history strategy. Previous research has found that life history strategy not only influenced by early environment but also by individual personality (Figueredo et al., 2004, 2005). Individuals with higher level of the dark triad are more impulsive, exploitative, focused only on personal interests, more focused on immediate benefits, and desire direct procreative-related benefits (Gladden et al., 2009). Individuals who experience more early adversity gradually develop an implicit attitude regarding the scarcity of resources and the brevity of life (Ellis et al., 2009). Thus, when individuals have difficult childhood environments, individuals with high dark triad choose fast life history strategy to obtain more resources (Jonason et al., 2014; Csatho and Birkas, 2018; Geng et al., 2021); whereas individuals with low dark triad have an advantage in delayed gratification and are good at careful planning (Jonason et al., 2010), which helps them to choose a slow life history strategy. Therefore, we propose:

*Hypothesis 3*: dark triad plays a moderating role in the association between childhood adversity and life history strategy.

In addition, dark triad may moderate the process of life history strategy on problem behaviors. Individuals who adopt fast life history strategies tend to pursue immediate benefits (Figueredo et al., 2004). The dark triad represents the dark side of personality, and individuals with high levels of the dark triad are more cynical, immoral, manipulative, and will do anything to achieve their goals, even if they do not conform to social norms (Jonason et al., 2015, 2017). Influenced by the high dark triad, individuals tend to do anything in their pursuit of immediate benefits and are more likely to have problem behaviors; therefore, individuals with the high dark triad will have more problematic behaviors when using fast life history strategies (Jones and Paulhus, 2010; Paulhus et al., 2022). In contrast, individuals with a low dark triad are more focused on the long term and are good at careful planning, which can effectively compensate for the lack of long-term consideration in the fast life history strategy (Paulhus and Williams, 2002). Therefore, individuals with a fast life history strategy show fewer problem behaviors under the influence of the low dark triad. One study found that the dark triad moderated the effects of fast life history strategy on aggressive behavior, and individuals who used fast life history strategies had significantly more aggressive behavior when they had higher levels of the dark triad (Shi, 2021). Therefore, we propose:

*Hypothesis 4*: dark triad moderates the association between life history strategy and problem behaviors.

### The current study

**Figure 1:**
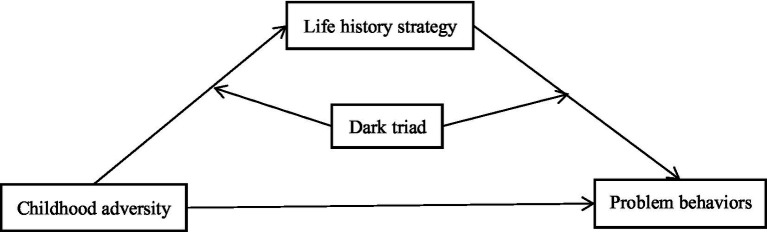
Proposed moderated mediation model.

To summarize, this study examined the combined effects of childhood adversity, life history strategy, and the dark triad on the problem behaviors (Figure 1), using new street corner youth on campus as participants and constructing a moderated mediation model to explore the mediating role of life history strategy and the moderating role of the dark triad in the relationship between childhood adversity and problem behaviors.

## Materials and methods

### Participants and procedures

To ensure that the results of the study were representative, data were collected from multiple schools. Considering the differences in the management methods of public schools and private schools, when selecting schools for research, they are also divided according to the nature of the schools. Among them, there are 8 junior high schools, including two private middle schools and six public middle schools; there are 4 senior middle schools, including one private high school and three public high schools; there are three secondary vocational schools, including two public secondary vocational schools and one private secondary vocational school; there are three colleges and universities, including one public school, two private schools, and one private undergraduate college. High schools develop knowledge-based students, while secondary vocational colleges develop application-based students who can go to work directly after graduation and have higher admission scores in high schools than in secondary vocational colleges.

The experimenter, as a psychology teacher, surveyed 19 schools over an 8-month period from April 2021 to December 2021 through semi-participant observation, interviews, and fieldwork. During this 8-month period, we mainly observed and interviewed on-campus students who interacted with off-campus street corner youth. During the survey, the experimenter purposefully selected new street corner youth on campus to complete the questionnaire. Our selection criteria for new street corner youth on campus were students who had interpersonal interactions with street corner youth, such as being friends with street corner youth, partying, etc. Before students answered the questionnaire, the experimenter would explain the precautions and read out the instructions. A total of 657 questionnaires were distributed, and 637 valid questionnaires were collected, with a valid return rate of 99.96%. The average age of the participants was 15.05 years old (*SD* = 0.94), with 429 males and 208 females. Data were collected in an anonymous manner after reading the instructions and signing the informed consent form, participants completed paper-version questionnaires within 15 min. After completing the survey, everyone was received a small gift. The authors’ university ethics committee approved the current research.

### Measures

#### Problem behaviors

Problem behaviors were assessed using the Strengths and Difficulties Questionnaire revised by Kou et al. (2007), which was measured in China and proved to be appropriate as an instrument for evaluation of problem behaviors in children and adolescents, worthy of promotion and use (Du et al., 2006), and was proven to have good reliability and validity (Zhang, 2019; Han and Sun, 2022). It is a 25-item questionnaire consisting of five dimensions (emotional symptoms, conduct problems, hyperactivity, peer interaction problems, and prosocial behavior), such as, “I often argue with others.” The average of the total scores of the first four dimensions is the problem behavior score, with higher scores indicating more problem behavior. Participants rated all items on a 3-point scale (1 = does not meet to 3 = fully meets), with questions 7, 11, 14, 21, and 25 scored in reverse. In this study, the Cronbach’s ɑ was 0.89.

### Childhood adversity

Childhood adversity was measured using the Childhood Environment Scale developed by Ellis et al. (2009), and revised by Chen and Chang (2016). The scale was validated into Chinese language (Zhang and Liu, 2022) and is a 14-item questionnaire consisting of two dimensions (hardship and unpredictability), such as, “How many times in your childhood did you seek medical attention for injuries from beatings?” Participants rated all items on a 4-point scale (1 = never to 4 = always). With higher scores indicating greater hardship and unpredictability of childhood adversity. In this study, the Cronbach’s ɑ was 0.97.

### Life history strategy

The life history strategy was measured using the Life History Strategies Scale developed by Fiueredo et al. (2006), and revised by Wang et al. (2017). Although the original version of the questionnaire has received some criticism in recent years (Gruijters and Fleuren, 2018; Manson et al., 2020), the Chinese version of the scale revised by Geng et al. is widely used in China and has good reliability (Zhang and Geng, 2021). The scale contains 20 items scored and is a uni-dimensional scale on a 7-point scale (1 = Completely disagree to 7 = Strongly agree), such as, “I avoid taking risks.” With a high score indicating a slow life history strategy. In this study, the Cronbach’s ɑ was 0.97.

### Dark triad

The dark triad was measured using the Dark Triad Scale developed by Jones and Paulhus (2014), and revised by Geng et al. (2015). The Chinese version of the scale revised by Geng et al. is widely used in China and has good reliability (Yang et al., 2021). It is a 12-item questionnaire consisting of three dimensions (Machiavellianism, Psychopathic, and Narcissism). Participants rated all items on a 7-point scale (1 = Totally disagree to 7 = Strongly agree), such as, “I do not care much about whether my actions are ethical or not.” With higher scores indicating higher predisposition to the dark triad personality. In this study, the Cronbach’s ɑ was 0.92.

### Date analysis

Descriptive statistics and correlations of key variables were analyzed using SPSS 26.0. The analysis of moderated mediation model was performed using PROCESS macro (Model 59). Prior to the analyses, the predictors were standardized. In all analyses, we included gender and age as a covariate.

## Results

### Common method deviation test

We addressed the possibility of common method variance, the results revealed that there are 10 factors with eigenvalues greater than 1, and the explained variance of the first factor was 24.68%, which is lower than the specified maximum of 40%. Therefore, common method variance was not a significant concern in this study.

### Preliminary analysis

The results of the descriptive statistics and correlation analysis are shown in Table 1. The scores of PB for new street corner youth on campus in this study were higher than the scores of adolescents in previous studies (*M* = 0.59, *SD* = 0.25), indicating that there were more problem behaviors for adolescents in this study than for the general youth (Liu et al., 2020). The scores of CA for the new street corner youth on campus in this study were lower than the scores of adolescents in previous studies (*M* = 17.97, *SD* = 0.34), indicating adolescents in this study did not have more difficult and unpredictable childhood circumstances compared to general youth (Sai et al., 2020). The scores of DT for the new street corner youth on campus in this study were higher than the scores of adolescents in previous studies (*M* = 20.51, *SD* = 12.08), indicating that the dark triad personality tendencies of adolescents in this study were higher than those of general adolescents (Gao et al., 2020). The scores of LHS for the new street corner youth on campus in this study were lower than the scores of adolescents in previous studies (*M* = 114.81, *SD* = 16.17), indicating that adolescents in this study adopt faster life history strategy compared to general youth (Sai et al., 2020).

**Table 1:** Descriptive analysis and correlations among main study variables (*n* = 637).

	*M*	*SD*	1	2	3	4
1.CA	17.16	6.96	1			
2.LHS	93.35	28.84	−0.27^**^	1		
3.DT	27.67	13.70	0.33^**^	−0.47	1	
4.PB	1.91	0.38	0.20^**^	0.12^**^	0.12^**^	1

CA, childhood adversity; LHS, life history strategy; DT, dark triad; PB, problem behavior.

* *p* < 0.05;

** *p* < 0.01.

The correlation analysis between the variables revealed that CA was negatively associated with LHS and positively associated with PB, these results are consistent with previous studies (White et al., 2013; Salo et al., 2021). LHS were positively correlated with PB, this result is inconsistent with previous study (Figueredo et al., 2004). DT was positively correlated with PB, this result is consistent with previous study (Li et al., 2016). CA was positively associated with DT, this result is consistent with previous study (Zhan et al., 2021).

### Testing the mediating role of life history strategy

First, model 4 of the SPSS macro program Process was used to test the mediating effect of LHS between CA and PB while controlling for gender and age. The results show (see Table 2 for details) that CA can significantly positively predict PB, CA can significantly negatively predict LHS; after incorporating LHS into the regression equation, CA can still significantly positively predict PB, and the direct effects of CA on PB and the relationship between LHS. The Bootstrap 95% confidence interval of the mediation effect does not contain zero, indicating that the partial mediating effect of life history strategy between CA and PB is significant (*ab* = −0.05, *SE* = 0.01, 95%CI = [−0.08, −0.02]). According to the research method of Wen and Ye (2014), when ab and c’ moved in opposing directions, it indicated that LHS had “suppressing effects” on the relationship between CA and PB, and the effect size is |ab/c’| = 18.39%. Overall, effect sizes are modest.

**Table 2:** Results of the mediation model.

Predictors	Model 1 (PB)		Model 2 (LHS)		Model 3 (PB)	
	*β*	*t*	*β*	*t*	*β*	*t*
Gender	0.05	1.39	0.10	2.70^**^	0.04	0.91
Age	−0.05	−1.26	−0.02	−0.52	−0.05	−1.18
CA	0.21	5.25^***^	−0.25	−6.50^***^	0.26	6.33^***^
LHS					0.19	4.65^***^
*R^2^*	0.05		0.08		0.08	
F	10.06^***^		19.51^***^		13.20^***^	

All variables in the model are standardized and brought into the regression equation, same below. *p < 0.05; **p < 0.01; ***p < 0.001.

### Testing the moderated mediation model

A Moderate Mediation Model of the SPSS macroprogram Process was used to test the moderating effect of DT, controlling for gender and age, and the results were found (see Table 3): the interaction term between CA and DT significantly and negatively predicted LHS; the interaction term between LHS and DT significantly and negatively predicted PB. Thus, DT moderated the first half as well as the second half of the pathway of LHS mediating CA and PB.

**Table 3:** Moderated mediation analysis.

Predictors	LHS		PB	
	*β*	*t*	*β*	*t*
Gender	0.11	2.87^**^	0.03	0.75
Age	−0.02	−0.57	−0.04	−1.05
CA	−0.21	−4.81^***^	0.13	2.87^**^
DT	0.84	2.05^*^	−0.01	−0.19
CA × DT	−0.07	−2.85^**^	0.03	1.10
LHS			0.10	2.06^*^
LHS × DT			−0.19	−4.27^***^
*R^2^*	0.10		0.13	
*F*	13.97^***^		13.00^***^	

*p < 0.05; **p < 0.01; ***p < 0.001.

**Figure 2:**
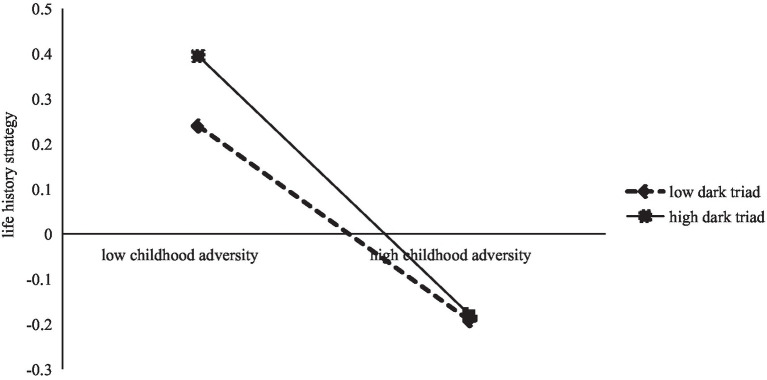
Life history strategy as a function of childhood adversity and dark triad.

**Figure 3:**
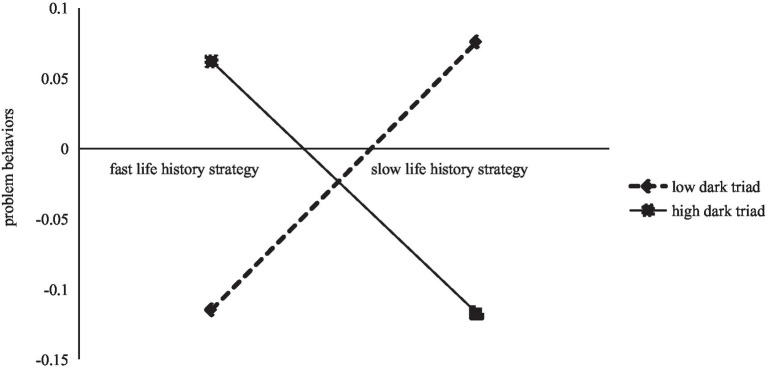
Problem behavior as a function of life history strategy and dark triad.

Simple slope tests were performed by taking the values of DT plus and minus 1 standard deviation. Figure 2 shows that the negative predictive effect of CA on LHS was significant when the level of DT was low (*β* = −0.15, *t* = −2.41, *p* < 0.05), and the negative predictive effect of CA on LHS remained significant when the level of DT was high, but the increase was relatively slowed (*β* = −0.29, *t* = −7.03, *p* < 0.001). Figure 3 shows that the positive predictive effect of LHS on PB was significant when levels of DT were low (*β* = 0.28, *t* = 6.43, *p* < 0.001).

## Discussion

In summary, this study examined the effects and mechanisms of childhood adversity, life history strategy, and the dark triad on the problem behavior of new street corner youth on campus. However, there continue to be shortcomings in this study. This study is a cross-sectional study, lacks strict attribution, and cannot make longitudinal comparisons. This study examines concurrent (not longitudinal data) data and that such data sets do not allow to define the direction of association among variables. In addition, the method of the questionnaire survey is used, and other survey methods are lacking. In view of the above limitations, attention should be paid to the use of multichannel data sources in the future research process to avoid the singleness of research methods.

This study found a significant positive correlation between CA and PB, supporting hypothesis 1. This is consistent with previous research findings that adolescents with adverse childhood experiences have more PB, such as fighting and truancy (Salo et al., 2021), which indicates that the more difficult and unpredictable the childhood environment of new street corner youth on campus, the more PB. The more varied or long-lasting early life misadventures increase the likelihood of experiencing risky, harmful, or negative events during growth (Ferraro and Shippee, 2009), and have serious negative effects on mental health (Evans and Kim, 2007). For example, early life misadventures increase the risk of later adverse habits such as smoking (Lloyd and Taylor, 2006), alcohol dependence (Lloyd and Turner, 2008), and obesity (Greenield and Maks, 2009), which in turn can have lasting adverse health effects.

In the results of the correlation analysis, we found a positive correlation between LHS and PB, which is not entirely consistent with previous results (Figueredo et al., 2004). We can provide an explanation for this. Firstly, although the results of this correlation analysis are not entirely consistent with existing theoretical speculations, they do not affect the validity of the moderate mediation model in the present study. Secondly, the problem behaviors of the adolescents in this study consisted mainly of emotional symptoms, conduct problems, hyperactivity, and peer interaction problems. There is no previous research directly demonstrating the relationship between LHS and PB of adolescents, but one study found that individuals who use slow life history strategy also have some emotional problems, only to a lesser degree and for a shorter duration (Zhao et al., 2019). From this result, we also deeply realize that the mechanism of action between LHS and PB deserves further research.

In addition, it was found that LHS mediated the relationship between CA and PB, that is, CA can not only directly affect PB, but also indirectly affect PB through LHS, supporting Hypothesis 2. This study mainly understands CA from two aspects: hardship and unpredictability. Individuals in hardships will face many unfavorable factors. Morbidity and mortality are the core indicators to measure hardship. Unpredictability refers to the future unpredictability. Evolutionary psychology suggests that external environmental conditions influence the choice of individual LHS. The arduous and unpredictable life circumstances cause individuals to fear that long-term effort will not be reported, so they prematurely devote life resources to reproductive tasks rather than focusing on long-term benefits, and tend to develop themselves rapidly and resort to manipulating others to benefit (Belsky et al., 2012). Individuals who adopt a fast LHS start sexual behavior earlier, are more impulsive and risk-taking, emphasize having fun in the moment, focus on immediate benefits at the expense of long-term development, and have more behaviors that violate social norms (Sng et al., 2017).

The present study found that the DT moderated the first half of the pathway of LHS mediating CA and PB, a finding that supports hypothesis 3, suggesting that DT diminishes the likelihood that new street corner youth on campus experiencing CA will choose slow LHS and promote the choice of fast LHS. Persons with different personality traits will adopt different LHS to adapt to the environment (Figueredo et al., 2004, 2005). When childhood environments are difficult and resources are scarce, individuals with a high DT are more selfish, focused on immediate benefits, and desire direct reproduction-related benefits in order to obtain more resources, accelerating individuals to choose fast LHS (Gladden et al., 2009). Thus, individuals with higher DT have a negative correlation between CA and slow LHS. In contrast, individuals with lower DT are good at delaying gratification (Jonason et al., 2010). In spite of difficult childhood environments and resource constraints, individuals with lower DT are able to plan rationally and focus on long-term benefits, so individuals with low scores on the dark triad have a negative association between childhood adversity and fast LHS (Jonason et al., 2014; Csatho and Birkas, 2018; Geng et al., 2021). Thus, DT moderates the relationship between CA and LHS.

DT moderates the second half of the pathway for the mediating effect of LHS on CA and PB, which supporting Hypothesis 4. The results of simple slope tests showed that slow LHS was positively associated with PB when the score of DT was low. This is because individuals with dark triad often unscrupulous and even non-normative in their pursuit of benefits and are more likely to have problem behaviors (Jones and Paulhus, 2010; Paulhus et al., 2022). Individuals with the low DT, although focused on long-term planning, are characterized by impulsiveness, apathy, and disregard for traditional morality, and even when individuals adopt slow LHS and focus on long-term planning, they may take some wrong approaches in the planning process, resulting in more problematic behaviors (Paulhus and Williams, 2002).

The results of this study have significant implications for the prevention of problem behavior among new street corner youth on campus. First of all, the childhood environment has laid the material and psychological foundation for the development of new street corner youth on campus. Families, schools, and all sectors of society should strive to optimize the growth environment of children. The impact of a bad childhood environment on children’s life is indelible. They should strive to create a stable living environment for children and reduce the occurrence of unpredictable events. Secondly, new street corner youth on campus should be guided to adopt a slow life history strategy, not focusing on current interests, but long-term interests, planning for future life, and rationally allocating and weighing their own living resources. Finally, we should pay attention to the personality traits of new street corner youth on campus, build a healthy psychological quality, establish a correct worldview, outlook on life and values, and cultivate a positive personality trait. There is hope for a fully adaptive life even when facing adversities.

## Conclusion

In summary, the current study suggests that CA is positively associated with PB. After controlling for gender and age of new street corner youth on campus, mediation analysis showed that LHS mediated the association between CA and PB. Moreover, moderated mediation analysis further indicated that DT moderated the association between CA and LHS, as well as the association between LHS and PB.

## Data availability statement

The original contributions presented in the study are included in the article/supplementary material, further inquiries can be directed to the corresponding author.

## Ethics statement

The studies involving human participants were reviewed and approved by Ludong University Ethics Committee. Written informed consent to participate in this study was provided by the participants’ legal guardian/next of kin.

## Author contributions

LLF: conceptualization, methodology, formal analysis, resources, data curation, and writing—original draft. WJM: conceptualization, methodology, validation, and writing—review and editing. All authors contributed to the article and approved the submitted version.

## Conflict of interest

The authors declare that the research was conducted in the absence of any commercial or financial relationships that could be construed as a potential conflict of interest.

## Publisher’s note

All claims expressed in this article are solely those of the authors and do not necessarily represent those of their affiliated organizations, or those of the publisher, the editors and the reviewers. Any product that may be evaluated in this article, or claim that may be made by its manufacturer, is not guaranteed or endorsed by the publisher.
